# Navigating the design of simulated exercising peers: insights from a participatory design study

**DOI:** 10.3389/fdgth.2025.1551966

**Published:** 2025-05-12

**Authors:** Alessandro Silacci, Mauro Cherubini, Maurizio Caon

**Affiliations:** ^1^ Persuasive Technology Lab, Information Systems Department, University of Lausanne, Lausanne, Switzerland; ^2^ Digital Business Center, School of Management of Fribourg, HES-SO University of Applied Sciences and Arts Western Switzerland, Fribourg, Switzerland

**Keywords:** AI agent, participatory design, physical activity, self-determination theory, well-being, young adults

## Abstract

**Background:**

To fight sedentary lifestyles, researchers have introduced various technological interventions aimed at promoting physical activity through social support. These interventions encourage people to exercise together, maintaining high levels of motivation. However, the unpredictable nature of human peers makes it challenging to control behavior and balance these interventions effectively. Artificial intelligence agents, on the other hand, can provide consistent social support and are more controllable. Hence, we propose Simulated Exercising Peers (SEPs) as a promising solution for providing agent-based social support for physical activity.

**Method:**

Participatory design sessions were conducted, involving young adults in the creation of SEP-based interventions. Sixteen participants generated four prototypes that varied in aesthetics, behavior, and communication style, with outcomes analyzed through the lens of Self-Determination Theory to better understand the motivational implications of each design.

**Results:**

Findings highlight key components crucial for designing SEPs that enhance acceptance and efficiently integrate into physical activity interventions. Additionally, the study revealed how the aesthetics and behavior of SEPs could potentially deceive users, which can lead to user disengagement from interventions involving SEPs. Participants also defined two distinct social roles for the SEPs, i.e., coach, and companion, each associated with unique communication styles.

**Conclusion:**

This study offers five design guidelines for the development of SEPs, AI agents aimed at promoting physical activity through social support, and highlights opportunities for their integration into broader physical activity interventions.

## 1 Introduction

Engaging in regular exercise sessions of moderate-to-vigorous intensity can have positive effects on human health and overall well-being. However, according to a 2022 World Health Organization (WHO) report, more than 1.4 billion people (i.e., more than a quarter of Earth’s population) do not meet the recommended physical activity levels (1). WHO’s report also highlights that physical inactivity will represent a yearly cost of 27 billion US dollars in disease treatment between 2020 and 2030.

Physical activity does not exhibit a linear decline across all age groups. Rather, researchers have identified adolescence and young adulthood as crucial periods when individuals’ physical activity habits undergo significant changes (2–4) and bad habits might crystallize (5).^1^ Hence, young adulthood becomes a pivotal period for promoting long-term physical activity behaviors within this demographic (6), as changes during this period are likely to persist into adulthood.

Studies have highlighted that male adolescents and university students mention the absence of social support as a barrier to engage in more physical activity (7–9). To address such barriers, Human-Computer Interaction (HCI) research leverages persuasive digital technologies to promote behavior change, highlighting the crucial role these technologies play in enabling interventions based on various psychological theories (10). In particular, several interventions have demonstrated the effectiveness of promoting peers social support in increasing engagement in physical activity (11–15). One of the most obvious advantage of these interventions was the ability to connect peers who might be interested in exercising together, but who could not be available at specific times or places.

However, these seminal studies have also revealed the limits of these approaches. Firstly, even if technology could bridge the gap of finding an exercising partner, several users might still be reluctant to engage in physical activity due to self-consciousness and fear of being judged (16). Also, it is hard to match people effectively. Exercising with someone who might be *over-* or *under-performing* might prove disengaging over the long run (17). This highlights how individuals’ idiosyncratic preferences make it complex to predict the effectiveness of these interventions as individuals might be more receptive to certain kinds of partners (e.g., family, friends, strangers) (18), or social strategies (e.g., competition, cooperation), as studied by Orji et al. (19).

Given the challenges in designing interventions that could scale to large populations of users, researchers in HCI have introduced the use of agents, i.e., computer-based entities that can act autonomously and interact with users. AI agents have the advantage that they are always available and that they can adapt their appearance [e.g., animals (20, 21), humans (22)], behavior, and communication abilities (23) according to the user preferences. The main objective of these agents is to understand and assist individuals in their physical activity, providing their human peers with tailored feedback, instructions, education, and a social presence (24). In this paper, we are going to refer to this type of agents as Simulated Exercising Peers (or SEPs, in short). Unfortunately, the design of SEPs remains largely undefined. Their effectiveness heavily depends on their ability to establish a relationship with individuals, provide relevant and context-sensitive feedback (25, 26), and create meaningful social connections (20, 27–29). Previous research on agents for physical activity focused on conversations, and has overlooked the potential implications of the agents’ visual characteristics in physical activity interventions (30). Unfortunately, to this day, there are no guidelines to be found in the scientific literature that can help designing SEPs. These studies failed to develop specific design recommendations for SEPs. Most importantly, prior research has designed interventions *deductively*, moving from psychological theories of human motivation into design embodiments.

This study aims to address current gaps in drafting design guidelines for SEPs by exploring aesthetics, behavior, and communication aspects of these agents. Contrarily to prior work, we aim at generating these recommendations *inductively*, using a bottom-up approach. This approach could greatly benefit the field and the specific design space of SEPs by revealing nuanced considerations in design (31). Therefore, we conducted a participatory design study: a collaborative approach to design that actively involves stakeholders—especially end-users—throughout the design process to ensure the resulting technologies or solutions align with their needs, values, and contexts (32). We involved 16 university students (19–28 y.o.) ensuring participants’ background heterogeneity (33). Participants were asked to co-design their ideal SEP specifically focusing on their visual aspects, their behavior, and communication style. To analyze the solutions produced by the participants, we decided to use the Self-Determination Theory (34). This theory is particularly useful in studying SEPs as one of its main constructs is *relatedness*, namely the need to feel connected to other people in a meaningful way (35), which is a fundamental aspect of the SEPs’ mission. Also this theory has already been scientifically validated through technology-based empirical studies (36–39).

As a first contribution of this study, we provide an in-depth analysis of the requirements and expectations of young adults regarding AI agents designed to support their daily physical activity. Our work emphasizes three core aspects crucial for creating believable and relatable agents: emotional intelligence, personalization, and contextual relevance. Specifically, our research extends the concept of agent believability from virtual worlds literature, demonstrating that an agent’s visual appearance significantly influences user expectations of its behavior and communication skills (40–42). Through participatory design, we discovered that integrating SEPs’ behavior with their aesthetics is crucial for creating engaging and motivating agents to support physical activity. Unlike previous studies on relational agents (43) and virtual coaching (44), our participants envisioned SEPs as entities sharing the physical activity journey with users as peers rather than acting as third-party advisors.

Secondly, this study reveals the critical impact of *deception* on SEPs, which can arise from misleading aesthetics, behavior, and communication skills of virtual agents. The young adults defined deception as believing that a user is a human being when it is actually an AI agent. To this end, the implications of this study suggest avoiding human-like appearances for SEPs, and preferring animal or cyborg aesthetics instead. Participants highlighted that deception not only undermines trust but also discourages engagement and induces an eerie feeling. Additionally, our study highlights the challenges of creating human-like agents, as users expect higher levels of believability, leading to frustration (45) and uncanny feelings when these expectations are not met (46).

Thirdly, our work provides actionable guidelines for designing SEPs that foster basic psychological needs, a key concept in SDT for promoting intrinsic motivation, which is the strongest type of motivation. Our findings emphasize the importance of relatedness, or social connectedness, identifying that SEPs can motivate participants by establishing mutual care relationships. This completes the existing literature as it only typically explores one-sided care relationships (22, 28, 47).

Finally, this paper provides specific and practical design implications for developing AI agents that effectively support physical activity and promote overall well-being, filling the gap in the fields of HCI and behavior change technologies.

## 2 Background

Behavior change theories provide a framework for understanding and influencing how individuals alter their behaviors, making them essential for developing effective interventions (48) in different fields. The use of these theories has been highly increasing in the context of physical activity—notably since the WHO expressed alarming concerns on the detrimental effects of physical inactivity and sedentary behavior (1). The landscape of behavior change theories in the context of physical activity is broad and continually expanding (49, 50). This growth makes it challenging to determine which of the theories is best suited for a given intervention. Relying on theoretical frameworks is essential for assessing and predicting the impact of technologies and features on behavior change (51). Additionally, employing behavior change theories can facilitate the development of interventions that have long-term effects on individuals’ behavior (52). Integrating these theories is even more important in the context of the third-wave of HCI (53), opening new horizons for technology supported behavior change research (51, 54).

### 2.1 Behavior change theories in the context of physical activity

Although a broad range of theories to explain human behavior exists, researchers often focus on a narrow selection when investigating physical activity promotion (55, 56). These predominant theories include the Social Cognitive Theory (SCT) (57), the Transtheoretical Model (TTM) (58), the Theory of Planned Behavior (TPB) (59), and the Self-Determination Theory (SDT) (34).

SCT, as introduced by Bandura (57), emphasizes the significant influence of social and environmental factors on individual behavior. This theory suggests that behavior is learned by observing others within a context of continuous interaction among environmental, behavioral, and personal cognitive factors. Central to SCT is the concept of self-efficacy, which is an individual’s belief in their ability to succeed in specific situations. Self-efficacy is shaped by four main sources: *mastery experiences*, where successes and failures respectively strengthen or undermine personal efficacy beliefs; *vicarious experiences*, where observing peers succeed can enhance one’s belief in their own abilities; *social persuasion*, where verbal encouragement from others persuades individuals of their capability to succeed; and *somatic and emotional states*, where individuals rely on their physical and emotional conditions to judge their capability in achieving a given activity. In SCT, self-efficacy, alongside personal goals, is a critical determinant of physical activity behaviors, as highlighted in the literature (60). However, the application of SCT in addressing physical inactivity has yielded inconsistent results, particularly concerning the impacts of outcome expectations and socio-structural factors (61, 62). Moreover, the effectiveness of SCT varies with the age of participants, with older individuals generally showing more positive behavioral changes. Due to these mixed findings, we decided against basing our study on SCT.

TPB rather posits that an individual’s intention to perform a behavior is the primary predictor of actual engagement in that behavior (59). This intention is influenced by three key factors: *attitude towards the behavior*, *subjective norms*, and *perceived behavioral control*. Attitude refers to one’s evaluation (either positive or negative) of performing the behavior, subjective norms is defined as the perceived peer pressure to perform or not the behavior, and perceived behavioral control represents an individual’s belief in their capabilities to execute the behavior. Despite the significance of these factors in shaping intentions, research indicates that even substantial changes in intentions often lead to only modest changes in actual behavior (63). This gap highlights a limitation of TPB: it does not incorporate volitional strategies such as planning and self-regulation, which are crucial for behavior maintenance (55, 64). Additionally, TPB primarily focuses on the initiation of behaviors rather than their sustained execution and lacks comprehensive longitudinal studies to effectively differentiate between individuals who maintain behaviors and those who do not (65). Given these limitations, particularly the theory’s inadequate focus on long-term behavior maintenance, we decided not to use TPB as a framework for designing SEPs, which aim to promote sustained physical activity.

TTM offers a structured, six-stage approach to understanding behavior change, which includes a focus on maintenance, unlike TPB (58). TTM outlines a progression through distinct stages that reflect an individual’s readiness to change. The initial stage is *pre-contemplation*, a stage where individuals’ are not willing to change their behaviors. Whereas, individuals in the next stage, *contemplation*, are considering making a behavioral change but have not taken any actions yet. *Preparation* is the first intentional stage, where individuals have started taking actions towards changing their behavior, for example by increasing their daily physical activity levels. This stage is reached after regular repetitions of their actions for at least six months. When the actions keep to be repeated regularly for a longer period (i.e., six months or more), individuals are considered to be reaching the *maintenance* stage. TTM acknowledges that progression through these stages is not necessarily linear but can be cyclical, with individuals potentially moving back and forth between stages. Despite its structured approach and inclusion of a maintenance stage, TTM faces criticism for several reasons such as the lack of evidences on the positive effects of this theory (66), and the lack of validated algorithms to assess the individuals’ current stage of change (67). More importantly, TTM does not account for key external factors that could influence individuals’ behavior (68) such as the social factors (69), a crucial element of SEPs’ design. Given these limitations, particularly the model’s insufficient consideration of external social factors fundamental for SEPs’ design, TTM, while insightful, may not fully address the needs of designing effective SEPs.

SDT is a broadly applied theory in technological behavior change interventions that do not necessarily target physical activity (70); however, research relying on it has been consistently growing (71). SDT is an organic theory composed of multiple sub-theories, enabling the application of its constructs at different stages of the interventions, demonstrating the high versatility of this theory. These observations have motivated us to use SDT as a foundational theory for designing SEPs, AI agents specifically designed to support individuals’ physical activity and foster relatedness.

### 2.2 Self-determination theory applied to physical activity support

SDT offers an insightful framework for understanding motivation through its organic and dynamic structure, which is articulated by multiple sub-theories (34). According to SDT, motivation moves along a continuum (35) going from amotivation to intrinsic motivation. The first state, amotivation, defines the reluctance or disinterest in the task or activity. Then, extrinsic motivation, is a state where the individual’s motivation depends on external stimuli and is further decomposed into multiple types, ranging from external: regulation to the task or activity driven by the external stimuli, to self-regulation: where individuals feel as the owners and the initiators of the task or activity. The continuum ends with intrinsic motivation, where individuals perform a task out of self-interest and their own volition. Interventions seek to support the transition of individuals from an extrinsically motivated behavior to an intrinsically motivated one.

Furthermore, Ryan and Deci (34) emphasize that reaching self-regulated motivation depends on satisfying Basic Psychological Needs (BPNs). Individuals are likely to take on activities that best promote the three BPNs: *autonomy*, *competence*, and *relatedness*. Autonomy is satisfied when an activity is performed out of self-interest and volition. Competence relates to believing in one’s ability to successfully accomplish a task or activity. Relatedness involves feeling cared for, caring for others, and being connected with others. Feeling that a peer is genuinely interested has a crucial effect on relatedness. Specifically, researchers have observed that the level of attachment to the care provider influences the promotion of relatedness (18). The feeling of relatedness is not limited to direct relationships and can appear online. For example, researchers have demonstrated the positive effect of social media health platforms on relatedness (72). Similarly, positive and encouraging comments have been observed to increase engagement and the promotion of relatedness in behavior change activities (15).

Autonomy is tied to the freedom of choice available to individuals, and relatedness hinges on the supportive presence of peers during tasks; however, fostering a sense of competence remains a challenging task. Therefore, SDT introduces the concept of *optimal challenge* as a construct to support the BPN of competence. Optimal challenge posits that a task should be neither too easy nor too difficult for individuals to perform; it should always be adapted to their capabilities (34, 73). By fostering competence, optimal challenge is likely to lead individuals to a feeling of mastery while performing a task and increase the chances of intrinsic motivation towards the task. Additionally, optimal challenge can facilitate experiencing *flow*—a state defining a complete immersion and focus into an activity—which leads to greater enjoyment and fulfillment (74).

The focus on competence within SDT exemplifies how its principles can be leveraged to enhance motivation and facilitate behavior change in technology-based interventions. SDT has been extensively applied and validated across various HCI domains related to behavior change (36). Specifically, in the realm of physical activity, SDT has deepened insights into motivational dynamics and behavior change processes in HCI interventions (71). However, a significant challenge identified in the literature is identifying optimal moments to provide support within fully integrated behaviors. This difficulty has prompted researchers to advocate for the integration of SDT principles early in the design phase of interventions, aiming to enhance the likelihood of creating impactful user experiences that effectively satisfy BPNs (75, 76). In response to this, taxonomies have been developed to assist in the design of HCI interventions, categorizing mobile app features based on the specific BPNs they target (77). Additionally, SDT principles have been incorporated into the design stages of interventions, proving beneficial in structuring persona designs (78) and in formulating design guidelines for conversational agents that support BPNs fulfillment (79).

Our decision to utilize SDT in designing SEPs is informed by these empirical findings and the theory’s applicability in exploratory contexts like ours. At this stage, we employ SDT as a framework to interpret key literature insights for designing SEPs that promote physical activity through social support. Further discussions will elaborate on how SDT’s constructs are integrated with SEPs, as they appear in our findings.

## 3 Related work

Designing virtual agents requires three things: a. defining the *visual characteristics* of the agent; b. establishing the *behavioral* guidelines, describing what it can or cannot do within the scope of the interaction with its human counterpart; and c. defining the rules that govern how it should *interpret and respond* to human language. In this section, we are going to review past research that covered these three areas.

### 3.1 Aesthetics of agents

The visual appearance of agents is a pivotal factor in user interaction, making it an essential consideration in the design of SEPs. Research distinguishes between two main types of supportive agents for physical activity: physical and virtual agents (80). Both types are capable of engaging in social interactions with humans through dialogues, natural cues like gestures, and emotional expressions (81). Studies indicate that both young and older adults tend to enjoy interactions with physical agents more, attributing a greater sense of presence to them compared to their virtual counterparts (82, 83).

Despite their advantages, physical agents come with higher costs, require specific materials, and need controlled environments for safe operation, which restricts their mobility and usability. These constraints make virtual agents, particularly those integrated into mobile interventions, a more viable option due to their pervasiveness and flexibility. Virtual agents allow for precise control over their characteristics, such as emotions, gestures, and visual appearance, enabling diverse aesthetic representations including dogs (20, 27), dragons (28), fish (84), and abstract creatures (29).

Moreover, the ability to personalize these virtual agents has been demonstrated to significantly boost user engagement (85, 86). This adaptability and personalization potential make virtual agents particularly suited for SEPs, offering a dynamic and user-centric approach to supporting physical activity.

The digital form of agents can also be used to provide feedback on users’ physical activity engagement by modifying their shape (e.g., making them wider or thinner) (20) or displaying specific emotional states (e.g., sick, unhappy) (28, 84). These emotional displays can directly impact users’ feelings of guilt (84), motivating them to engage in physical activity as they care for their agent.

Despite these findings, many studies offer limited justification for their visual appearance choices, making it difficult to assess their long-term impact on behavior change. This is concerning, as research in virtual worlds highlights that aesthetics play a crucial role in shaping human-agent relationships. Users form expectations about an agent’s behavior and capabilities based on its visual appearance (87, 88). In video game research, this is examined through the concept of agent believability—the perceived contrast between user expectations and the agent’s actual behavior (41, 42, 89). Significant discrepancies can undermine believability, negatively affecting user experience, immersion, and ultimately, motivation. However, current research on the believability of agents in physical activity contexts remains insufficient to provide definitive design guidelines for SEPs.

In addition to limited research on agents’ visual appearance, their design is further complicated by the need to accommodate idiosyncratic preferences, which can lead to unmet expectations. Video game researchers address this challenge by enabling players to personalize their avatars, allowing them to tailor avatars to their liking. Empirical studies show that personalization features strengthen users’ psychological connection with digital entities (90–92), increase attachment (93), and enhance engagement by supporting BPNs (85, 86). These findings reinforce the relevance of SDT as a foundational theory for SEP design.

Given the importance of aesthetics in shaping user interactions and motivation, it is crucial to investigate users’ expectations regarding the visual characteristics of SEPs. Despite a large interest for agents’ visual appearance in the video game literature, there are no existing studies that adequately inform the effects of visual appearance on promoting motivation in physical activity interventions. To address this gap, we aim to answer the following research question:

**RQ1**. What are users’ expectations in terms of visual appearance for a simulated exercising peer that can promote motivation in physical activity interventions?

### 3.2 Agent behavior

The use of agents as peers in group-based physical activity is a novel area with limited research on their specific behaviors. While extensive research has investigated the benefits of group-based physical activities involving human peers, the behaviors exhibited by agents in these contexts remain unclear. Grouping peers can foster the BPNs of autonomy, competence, and relatedness if implemented correctly (94–96). However, the success of group-based physical activity heavily relies on the behavior of each peer, as the idiosyncratic nature of individuals’ training habits and performance levels complicates the creation of interventions with appropriate social strategies. This can lead to unfair social comparisons. Consequently, agents have the potential to promote physical activity by adapting their behavior to individual preferences, but their behavior must align with users’ expectations based on their visual appearance.

To define agent behavior effectively, it is essential to consider the context of interaction, particularly the social strategies of competition and cooperation. These strategies can significantly influence the dynamics of promoting physical activity in group interventions (97). In competitive settings, individuals aim to outperform their peers, which has been observed to enhance individual performance (98). Conversely, in cooperative settings, individuals work together towards a common goal (99). Both strategies can lead to positive outcomes in physical activity interventions, depending on the social interdependence of the task (100).

Social interdependence exists when individuals share common goals, and their outcomes are affected by their own and others’ actions (101). For example, highly interdependent tasks (e.g., team sports such as football, volleyball, or basketball) require cooperation from the entire team, while tasks with low interdependency favor competition (e.g., fencing, tennis, or activities that can be done alone). Tauer and Harackiewicz (102) suggest using intergroup competition as a hybrid solution, benefiting from cooperation within a group and competition against another group, an approach known as “coopetition” in business and industrial strategy (103, 104).

However, group-based activities can present risks when rewards are contingent upon performance, leading to social comparisons where individuals compare their results with those of their teammates (105). Social comparison naturally occurs when tasks are tied to contingent goals and the intervention provides means for comparison (34, 106). Individuals may engage in upward comparison (comparing themselves with higher-performing peers) or downward comparison (comparing themselves with lower-performing peers) (107). The literature is unclear on the positive effects of social comparison, as it can undermine motivation if individuals consistently lose against their peers (34, 108). For example, research has shown that students frequently exposed to social comparison when comparing exam results may experience decreased motivation, particularly among underperforming students (109). To mitigate the negative effects of social comparison, designers should carefully consider the adopted social strategy and user behavior.

Determining the most suitable social strategy for SEPs remains challenging, as existing empirical results show both advantages and disadvantages for each strategy. If not properly addressed, SEPs’ behavior could impact social comparison and lead to disengagement from the intervention.

Another crucial factor in determining agents’ behavior is the concept of believability, which was also discussed in the previous subsection on aesthetics. Ensuring the believability of agents requires that their behavior is consistent with their visual appearance (41, 42, 89). Lankoski and Björk (87) categorize design patterns that define believable human agents in video games, noting that agents should have their own agendas and self-awareness of their environment. Video games often use agents as companions to assist players in tasks and guide them through environments (40). These agents need specific behaviors while still assisting users, and unpredictable behaviors can disrupt the predictability of AI agents’ messages or actions (110). Bailey and Katchabaw (111) proposed a framework for designing psychosocial behavior agents based on emergent gameplay in video games, suggesting that each agent possess its own goals and interests, influenced by user interactions.

While these elements are commonly associated with video games and virtual environments, they suggest that SEPs could have their own objectives, such as achieving a specific number of steps, while helping their human counterparts. This insight contributes to the development of believable agents that could enhance behavior change through SEPs. The literature also underscores the effectiveness of group-based physical activities and the significance of social strategies tailored to different task types. However, social comparisons, inherent to these strategies, can have varying impacts on performance, depending on how well they align with individual capabilities and preferences. To effectively design these interactions, it is crucial for designers to balance social comparison by considering individuals' activity levels and unique preferences. For SEPs, this opens avenues to introduce optimal challenges and position them as ideal competitors in small contests. The performance or difficulty level of SEPs might be adjusted through their visual appearance to ensure believability. Although existing empirical studies provide insights into how SEPs could facilitate interactions and offer support, most research on social comparison and strategies involves human interactions, and the believability of behavior in virtual agents remains less explored, particularly in contexts related to physical activity support. To bridge these knowledge gaps, our participatory design study aims to pinpoint user expectations concerning SEP behavior, focusing on the following research question:

**RQ2**. What are users’ expectations for the behavior of simulated exercising peers in promoting motivation for physical activity interventions?

### 3.3 Communicating with agents

In contrast with the aspects of aesthetics and behavior, the communication of agents for the promotion of motivation in physical activity interventions has been vastly investigated. Indeed, the use of Conversational Agents (CAs) became a main persuasive technique. This allowed us to identify the following traits as the most important ones to be integrated in the communication of SEPs, with reference to the current scientific literature.

CAs in the context of behavior change interventions are mainly implemented to deliver personalized advice (47, 112–117), provide support for users’ goal setting and attainment through accurate feedback (26, 118), and support users’ self-reflection on their behavior through educational support (114, 119, 120).

This set of cases illustrates the importance for CAs to support context awareness, and personalization to fit messages to users’ current situation. In the last two decades, researchers have been able to exploit various types of sensors embedded in wearables and smartphones to help understand users context and activities, facilitating the creation of meaningful and time-sensitive feedback (118). However, the design and creation of these messages remains a complex task for researchers, as demonstrated by op den Akker et al. (121)’s study resulting in a multidimensional framework to build motivational messages emphasizing on: the timing, the intention, the content and their representation. In addition to these dimensions, the CAs main leveraging point remains their conversational skills, which may require the use of experts to create and curate messages that fit the purpose of the behavior change intervention (26).

Through prolonged conversations with the agents, users tend to create a bond, a construct that can increase the persuasiveness of the CAs’ messages (47, 122). Notably, researchers demonstrated that long-term relationship with agents can exist and be maintained if both users and agents are engaged in the conversation (43). However, the design of relation-enabled agents complexifies designers’ work as they need to support certain conversational skills such as humor, social dialog, empathy, self-disclosure, and persistent memory (22). These implementation complexities are also illustrated by situations where CAs designed to support and coach for physical activity failed to provide interesting messages, and ran out of messages after some time (123, 124). These situations have mostly led users’ disengagement with their CAs and had negative effects on the users’ experience.

One of the main factors exacerbating user experience is agents’ failure to meet users’ expectations. Users’ expectations on agents’ communicative skills are often based on agent’s visual appearance, underscoring the importance of designing believable agents (125). For example, human-like avatars can lead users to develop higher expectations on agents’ ability to exhibit human qualities (126). Adopting human-like avatars and characteristics, though technically demanding, can enhance users’ sense of social presence (127). This is because demonstrations of humanness shape how users relate to and perceive the support provided by agents (45, 128).

Additionally, higher humanness can influence users’ feelings of autonomy, as such agents are perceived to make fewer errors and provide more predictable responses (79). However, mimicking human behavior without clarifying the agent’s non-human nature raises ethical concerns about *deception* (129). A risk that can arise when users are exposed to CAs with a high degree of humanness, potentially leading to “subtle deception” (128). Such deception can cause users to distrust the CAs and lose motivation in interacting with them (130).

In summary, CAs have the potential to provide a sense of social presence and support for physical activity. However, designing effective CAs remains a significant challenge, as research has yet to establish well-defined guidelines. A key dilemma lies in meeting user expectations, particularly when interventions utilize human-like avatars that heighten the perception of humanness. This often leads to a mismatch between user expectations and CA capabilities, resulting in frustration. Addressing this issue requires a deeper understanding of the specific communication skills users expect from SEPs—a topic explored in our participatory design study, guided by the following research question:

**RQ3**. What are the necessary features that would make users feel connected with simulated peers?

## 4 Methods

Through our related work, we identified gaps in the literature regarding agents’ characteristics that promote motivation in physical activity. Based on our review, we have formulated the following three research questions:

**RQ1.** What are users' expectations in terms of visual appearance for a simulated exercising peer that can promote motivation in physical activity interventions?

**RQ2.** What are users' expectations for the behavior of simulated exercising peers in promoting motivation for physical activity interventions?

**RQ3.** What are the necessary features that would make users feel connected with simulated peers?

Given the limited literature, we adopted a participatory design approach to explore specific needs and expectations for agents promoting physical activity, a method proven effective in enhancing well-being and fostering healthier lifestyles (131–136). We focused on students, a group prone to dropping physical activities due to significant life changes, engaging them in our design study.

Participatory design has addressed physical inactivity across various demographics including adolescents (137), individuals with autism (138), and older adults (133, 136). It has been instrumental in integrating behavior change technology (133), evaluating mobile interventions (137), and designing new physical activity interventions (135). A recent study by Janols et al. (139) combined participatory design with SDT to develop a virtual coach for older adults, identifying three motivational profiles, underscoring the value of this approach in understanding user expectations for SEPs’ aesthetics, behavior, and communication capabilities.

Our review also highlighted that the interaction with intelligent agents is influenced by factors like appearance (140, 141), personality (142), cognitive abilities (143), and proactivity (144), which vary by context and user characteristics. Designing the interaction by involving an AI agent with the target users allows infusing the users’ latent knowledge, culture and emotions into the artefact (145). This possibly increases the final user acceptance and trust. To encourage the ideation process, participants were involved in different activities like “group walk”, “co-design”, “focus groups”, and “mutual evaluation” that we detail in a later section (cf., Section 4.3).

### 4.1 Recruitment

Participants were recruited from the University of Lausanne’s participant pool, targeting young adults willing to engage in at least 45 min of walking and who owned a smartphone.

Invitations were sent to the entire participant pool two weeks before the first session, including a link to an online screener to verify eligibility for our study; details of this screener are available via the OSF repository dedicated to this study.^2^ Out of 111 registrants, 59 met the eligibility criteria, which required participants to be students available for at least one of the scheduled sessions. The screener also collected demographic information and assessed participants’ familiarity with physical activity tracking services.

In selecting participants, efforts were made to maximize gender diversity and accommodate varying availability during the experiment period, which consisted of two sessions across two different days. To enrich the discussions with diverse perspectives, we recruited students from various academic disciplines. The participant backgrounds included psychology (n=4), education science (n=3), computer science (n=3), criminology (n=2), medicine (n=2), politics (n=1), and Russian literature (n=1). Additional demographic details of the selected participants are provided in Table 1.

**Table 1:** The participants demographics, their assigned group, and the session they were participating to.

Participant	Gender	Age	Educational level	Study background	Session	Group
1	Female	23	Bachelor	Criminology	1	1
2	Male	21	Bachelor	Politics	1	1
3	Female	21	Bachelor	Russian literature	1	2
4	Male	20	Bachelor	Medicine	1	2
5	Female	18	Bachelor	Psychology	1	2
6	Female	19	Bachelor	Education science	1	1
7	Male	23	Bachelor	Medicine	1	1
8	Female	18	Bachelor	Psychology	1	2
9	Male	20	Bachelor	Education science	1	2
10	Male	19	Bachelor	Computer science	2	3
11	Female	28	Master	Psychology	2	3
12	Male	20	Bachelor	Psychology	2	4
13	Male	20	Bachelor	Computer science	2	4
14	Female	21	Bachelor	Education science	2	3
15	Female	20	Bachelor	Criminology	2	4
16	Male	19	Bachelor	Computer science	2	3

### 4.2 Participants

We selected 59 people from the respondents with a matching profile after the screening process. Participants were contacted via e-mail and registered for the sessions according to their availability. We recruited a total of N=16 participants (eight females). We grouped the participants according to their registered session day. During the sessions, we balanced gender representation in each session $N_{\mathrm{session}\;\mathrm{one}}=9$ (five females) and $N_{\mathrm{session}\;\mathrm{two}}=7$ (three females). Participants within a session were separated into two groups—the first day being composed of a group of four (two females) and another of five participants (three females)—while the second was composed of a group of four (two females) and a group of three (one female). Among the selected participants, 50% mentioned already having used a physical activity tracking application. Information on participant demographics and the sessions they participated to are further provided in Table 1.

### 4.3 Procedure

We reviewed the literature on participatory design studies to create our own process (146–151). Before conducting the study, we performed a dry run involving two, non-author, researchers to test and refine our study protocol.

**Figure 1:**
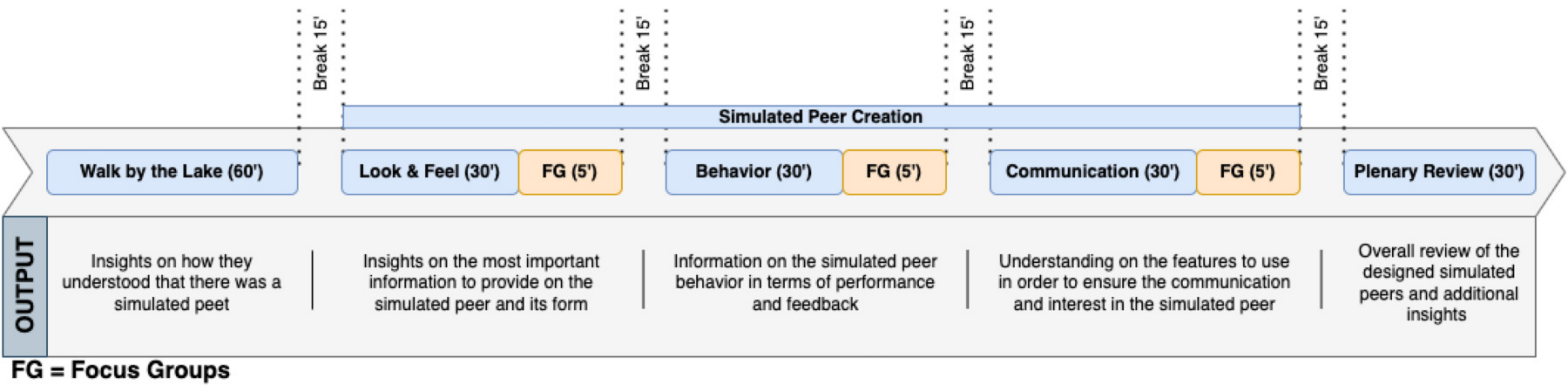
The timeline of activities for our participatory design study.

A detailed depiction of our process is shown in Figure 1. We organized the study into two sessions, each accommodating up to eight participants to facilitate management and adhere to social distancing protocols. Sessions were conducted on separate days to maximize attendance. Participants were divided into groups, engaging in identical activities concurrently. This setup ensured efficient management during the walk and simultaneous focus group discussions. All participants provided signed consent before sessions, which were conducted in French and audio-recorded. Compensation was set at USD 84 (CHF 75) for full participation.

The sessions included five main activities: *contextualization*, *co-creation: appearance*, *co-creation: behavior*, *co-creation: communication*, and *plenary presentation and discussion*. The *contextualization* phase introduced participants to SEPs via a team-based step tracking app, familiarizing them with the concept and their integration into mobile platforms. Each co-creation activity was followed by small focus groups to facilitate reflective discussion and ensure inclusive participation. These discussions helped monitor and guide the ideation process, ensuring engagement and balanced contributions across the groups. A dedicated slideshow supported each activity, with slides designed to prompt reflection on specific research questions related to SEPs (cf., Table 2). Further details and rationale for the study procedure are discussed in the following sections.

**Table 2:** The questions used to guide the design the SEPs for each stage of our participatory design study.

Design phase	Questions
Appearance	During the previous part (the walk) what particular elements helped you determine your group’s simulated peer?
	Which elements were visually essential? Which ones should be reproduced?
	What elements did you feel were missing?
	What would be the main visual characteristics of your simulated peer?
	How should it be represented? Ex: A drawing, an image, a 3D model, an object, etc.
	What form should the simulated peer take? Ex: Human/animal/robot/abstract form/something else?
Behavior	During the previous part (the walk), what particular elements helped you determine your group’s simulated peer?
	During the walk, what did you notice about your simulated peer’s number of steps?
	How did you feel during the walk as you watched its performance?
	How should a simulated peer behave when in your team? Ex: Have the same number of steps, vary the number of steps, be stronger/weaker, etc.
	How should it behave towards your performance? Ex: Encourage me when I don’t take enough steps, encourage me constantly, etc.
Communication	How would you communicate with your simulated peer?
	Referring to the walk, what would you have communicated with the simulated peer?
	What would make you want to communicate with your simulated peer?
	What relationship would you like to have with your simulated peer? How could this relationship be achieved?

#### 4.3.1 Contextualization

**Figure 2:**
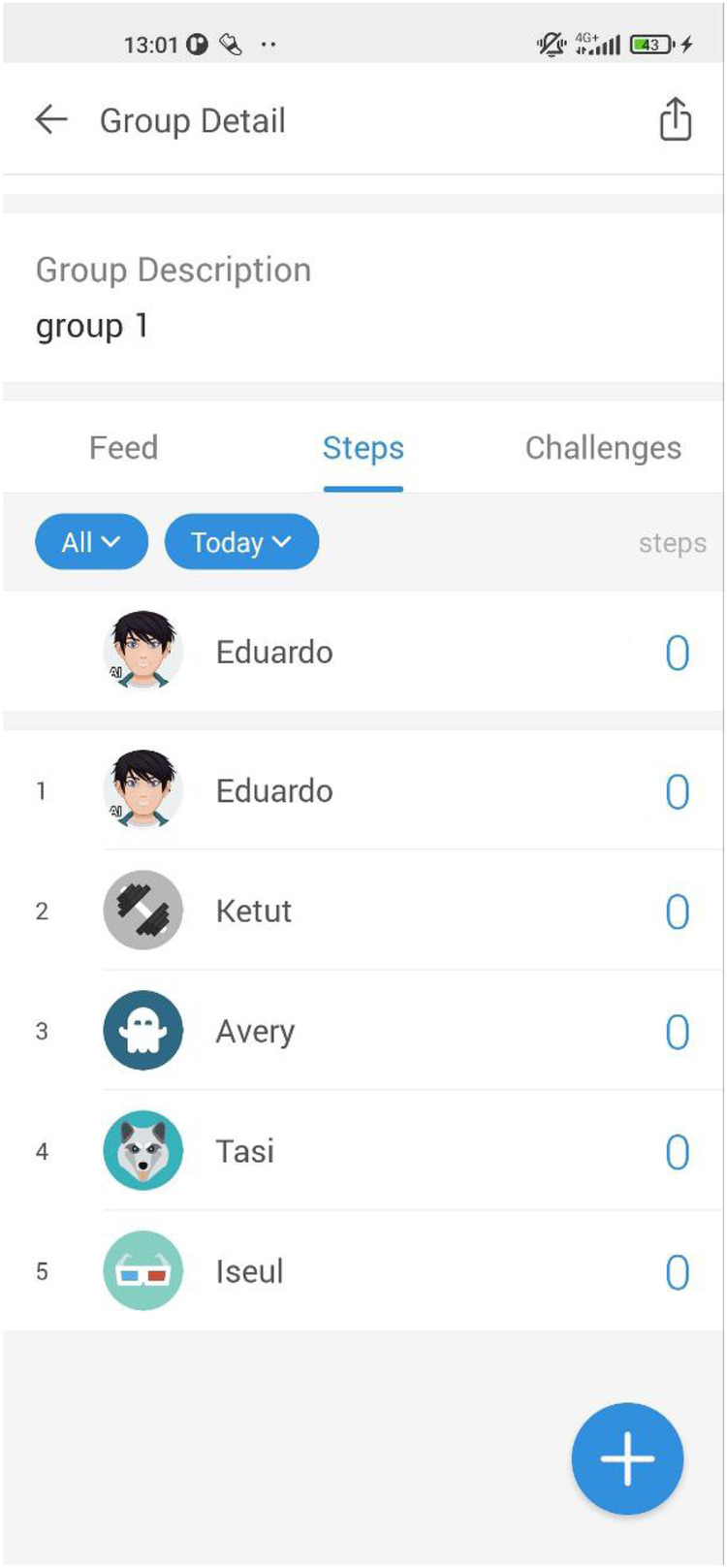
The *contextualization* phase’s starting situation with Eduardo our SEP and the rest of the first group. Screenshot from: Pacer app, Pacer Health, Inc.

**Figure 3:**
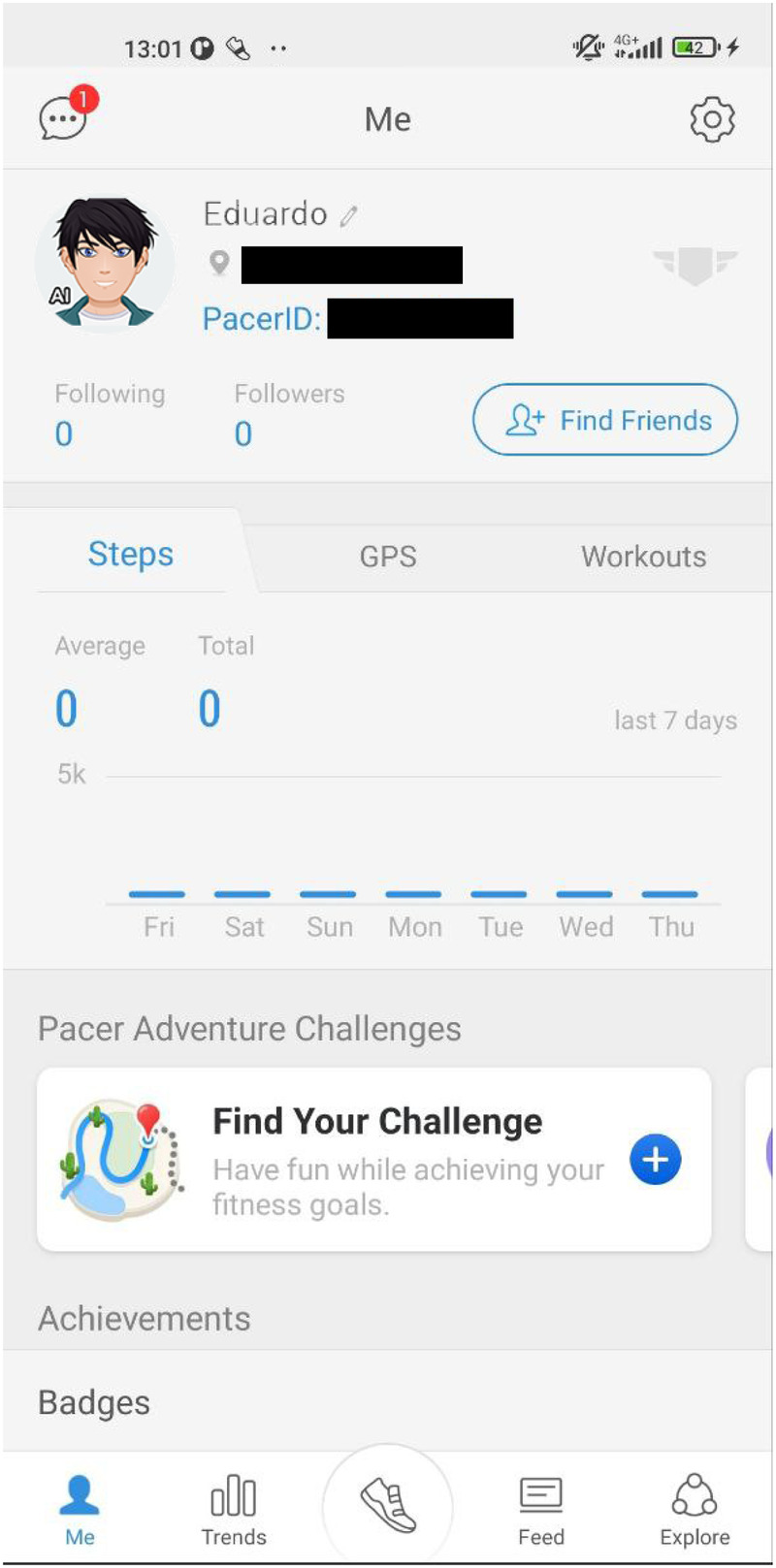
The profile of our SEP named Eduardo for the *contextualization* phase. Screenshot from: Pacer app, Pacer Health, Inc.

We conducted a 45-min walk by Leman's lake in Switzerland as a contextualization activity to spark ideation among participants, many of whom were unfamiliar with physical activity tracking apps and SEPs. Before the walk, introductions were made, and participants were given access to the Pacer app,^3^ chosen for its cross-platform availability, leaderboard, user profiles, and ease of use. All accounts were anonymized and subsequently deleted after the sessions. During the walk, our SEP, named “Eduardo”, was integrated into each group (cf., Figure 2), appearing as a user with an “AI” label on his profile picture (cf., Figure 3). Unknown to participants, Eduardo was present in both groups. Initially, Eduardo had no steps recorded but gradually began accumulating them. In the first 15 min, we encouraged participants to observe and discuss the scores and teams. By the second third of the walk, Eduardo had accumulated enough steps to appear on the team leaderboard and, in the final 15 min, increased his pace significantly, outperforming the participants. After the walk, we gathered feedback on the participants’ feelings and observations and clarified that Eduardo was not a real person but a SEP, explaining the underlying mechanism to prevent any deception.

#### 4.3.2 Co-Creation: appearance

For the ideation phase, participants were encouraged to co-create in groups using A3 blank paper sheets and various design tools such as pencils, colored pens, and post-its. The researcher facilitated the process by providing a series of guiding questions^4^ aimed at aiding the design process. In this initial co-creation session, participants focused on conceptualizing the appearance of the SEPs, defined broadly as their look and feel, to foster creativity without imposing restrictive expectations. Throughout the 30-min activity, the researcher and an assistant were on hand to offer additional information or prompt further reflection through targeted questions. Following the activity, a brief five-minute focus group discussion was held within each group to evaluate the designed SEPs and critically assess the proposed solutions.

#### 4.3.3 Co-Creation: behavior

Using the same material, participants had to reflect on the overall behavior of the SEPs. Iterating on their initial design, the groups had to add components and information about the behavior adopted by the SEPs. A set of questions was also prepared for this phase, to encourage discussion on the performance behavior of the SEPs. Additionally, we asked questions about the purpose of the SEPs and whether there was a link to its behavior.

#### 4.3.4 Co-Creation: communication

In the final design activity, participants were asked to consider methods of communication with their SEPs. Using the earlier walking activity as context, we explored their interest in interacting with the SEP, Eduardo. Specifically, we inquired whether participants wished to receive messages from Eduardo or had messages they wanted to convey to it. We provided prompts to guide discussions on the form of communication [e.g., text messages, kudos (152)], and its directionality. Participants were tasked with defining the purpose of these communications, such as offering encouragement or summarizing the activity. They were also instructed to incorporate these communication strategies into their earlier designs concerning the behavior and appearance of the SEPs. This activity lasted 30 min and concluded with a five-minute focus group discussion to review and refine their ideas.

#### 4.3.5 Plenary presentation and discussion

In this last activity, the participants were invited to present their work. Each group presented their creation. The groups first introduced their SEPs’ appearance, and provided a rationale for it. Then, they did the same for the behavior and finished with the communication. The other group was prompted by the researchers to comment and challenge the proposed design. Additionally, the participants were asked to explain how they envisioned the integration of their SEPs in a mobile application.

#### 4.4 Measurements

Voice recordings were made throughout the sessions to capture participant interactions. During the contextualization phase, researchers carried recording devices and moved towards participants during discussions, inviting them to speak closely to the recorders when asking prompting questions. In the design phase, each table was equipped with a recorder to document the participants’ thought and reflection processes. Focus groups were also thoroughly recorded, with devices strategically placed to ensure all participants were audible. In total, 8 h and 1 min of audio were recorded and subsequently transcribed across all sessions and activities. The transcription process was carried out by the two researchers, who also cross-checked each other’s work to ensure accuracy and consistency in the documentation.

### 4.5 Analysis

The participatory design was analyzed using the thematic analysis approach (153) after the transcription of the group discussions that were audio-recorded during the sessions. Transcripts were coded using the MaxQDA 2020.3 software. Two researchers worked together on the data analysis and identified 25 different codes. The coders blindly coded 10% of the transcriptions and reached a Cohen Kappa of 0.79, being considered as sufficient (154). Additionally, the coders reviewed the designs created during the sessions. A combination of the design reviews and the codes were then used to group the results according to SEPs: aesthetics, behavior, and social interaction. The codes resulting from our analysis are provided in Table 3.

**Table 3:** The results of the coding analysis, presenting the first order, second order and themes that emerged in the participatory design sessions.

First order	Second order	Theme
Effort Credibility	Credibility	Aesthetics
Initial Time		
Performance Explanation		
Simulated Peer Explanation		
Simulated Peer Form Perception		
Simulated Peer Integration		
Objective		Peer Appearance
Performance Projection		
Simulated Peer Form		
Simulated Peer Identification		
Simulated Peer Role		
Social Comparison		
Competition Feeling		Tailoring
Leaderboard User Position		
Performance Feeling		
Performance Reward		
Motivator		
Simulated Peer Tailoring		
Simulated Peer Behavior	Peer Purpose	Behavior
Simulated Peer Behavior Sentiment		
Simulated Peer Team		
Simulated Peer Care	Relatedness From Peer	Communications
Simulated Peer User Feedback		
Simulated Peer User Communication		
User Simulated Peer Communication	Relatedness To Peer	

### 4.6 Ethical considerations

The Institutional Review Board (IRB) of the University of Lausanne approved our study. Both the recruitment of participants and the participatory design sessions were carried out in October 2021. We received permission to have in-presence participatory design sessions with a certificate control and researchers were asked to ensure that all the participants had a valid COVID-19 certificate. The selected participants all provided their informed consent prior to the start of the experiment. Participants were free to address their colleagues with their pseudonyms instead of their real names during the sessions. In all our datasets, participants names were replaced by anonymous identifiers (e.g., P1, P2, etc.).

## 5 Results

The participatory design sessions yielded diverse SEP concepts, each embodying unique aesthetic, behavioral, and communicative traits. Participants envisioned SEPs in two primary roles: companion or coach, with each role fostering distinct relationships and expectations. For example, companion SEPs and users are expected to mutually care for each other, whereas coach SEPs are envisioned as more proactive in supporting human users.

A critical insight from these sessions was the potential for deception, where users might mistakenly believe an AI agent is human. This risk is especially pronounced in virtual environments with multiple digital entities and could lead to user disengagement if the AI’s nature is misperceived. To address this, participants suggested specific design strategies for SEPs’ aesthetics, behaviors, and communication styles to prevent such misunderstandings.

In the subsequent subsections, we delve deeper into these findings, categorizing them by SEPs’ aesthetics, behavior, and communication. For brevity, only the final designs are discussed here. Detailed sketches and data, shared in compliance with the transparency criteria outlined by Niksirat et al. (194), are accessible via an OSF repository.^5^

To ensure clarity in our results, group names have been abbreviated. Groups will be identified by the letter G followed by their number. For more details on the groups’ composition, refer to Table 1.

### 5.1 SEPs’ visual appearance

**Figure 4:**
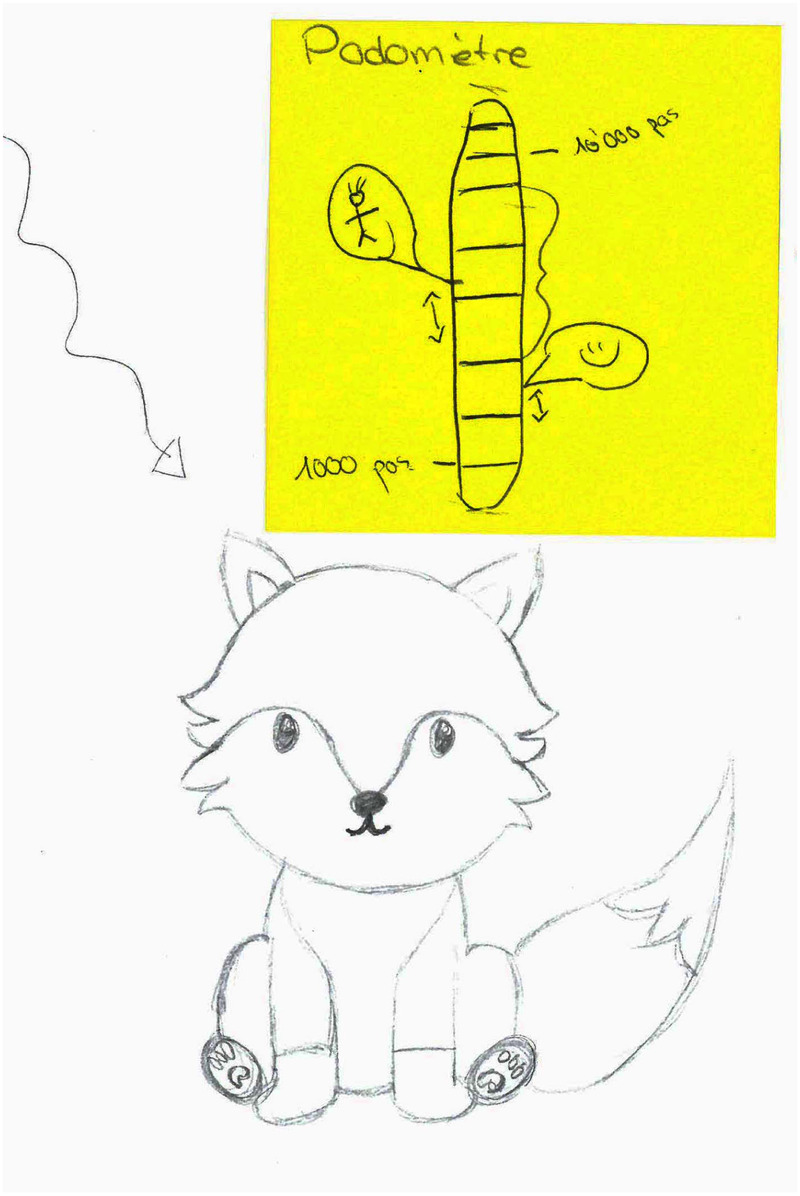
Group 1’s design representing a 2D animal (fox) and using a gauge as a performance comparison tool.

**Figure 5:**
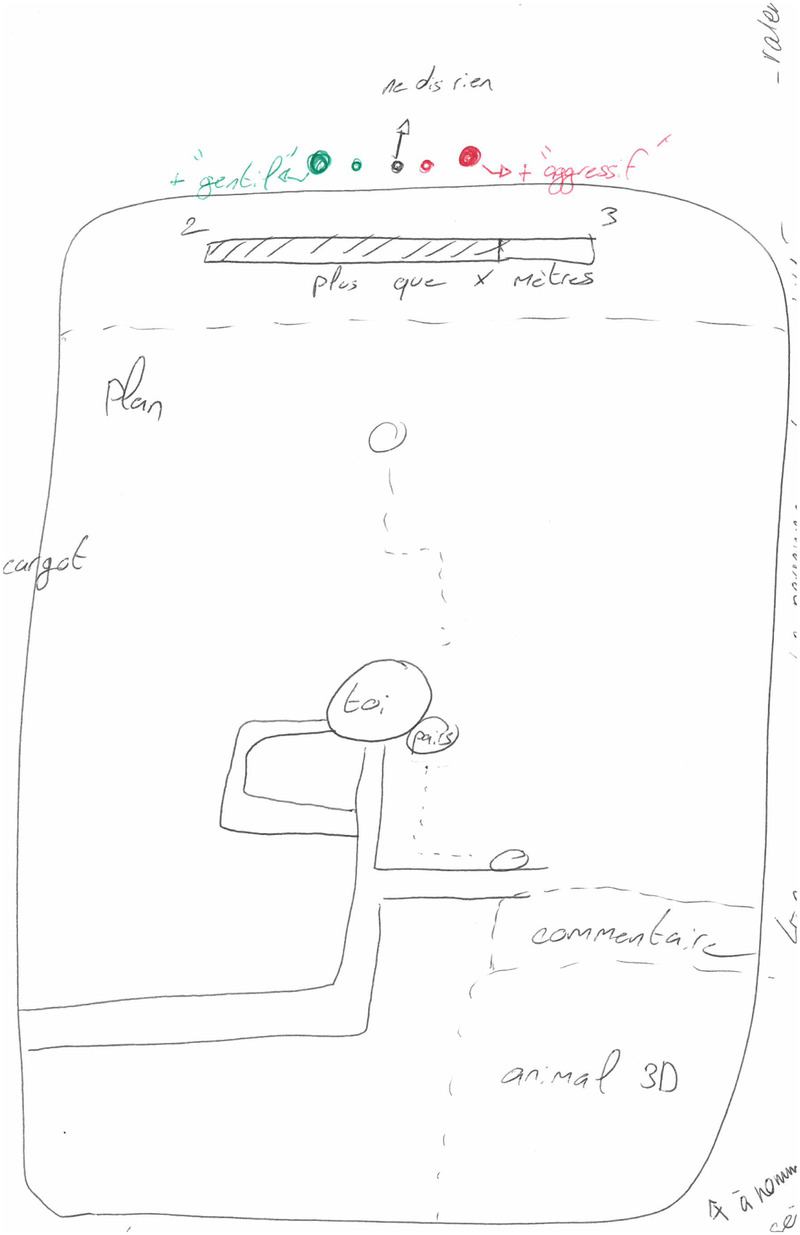
Group 2’s map display with the simulated exercising peer on the bottom-right, and on the side of the user’s position point. The mood from the simulate exercising peer is defined in the top, coloured, bar. Depending on the goal achievement rate, the simulated exercising peer would be more aggressive or remain kind.

**Figure 6:**
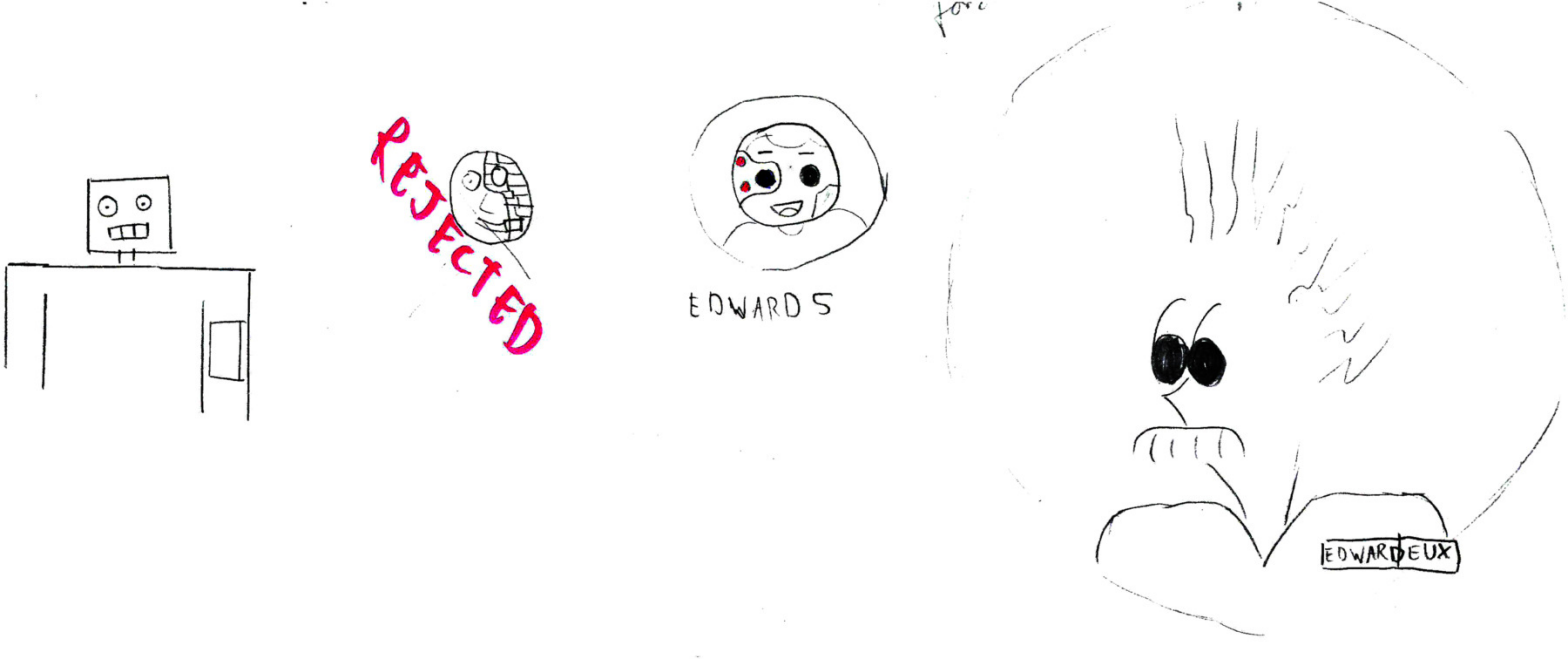
Group 3’s designs of a 2D simulated exercising peer, going from human-like (right) to a cyborg (middle). This group finally selected the cyborg (middle) representation.

**Figure 7:**
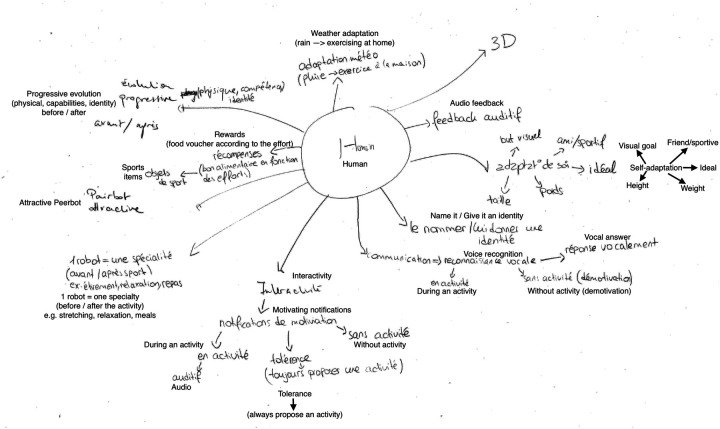
Group 4’s concept map of a human-like 3D simulated exercising peer taking the role of a virtual coach. The visual appearance of the simulated exercising peer should shape the current user on characteristics like height, and weight. The simulated exercising peer would represent an attractive version of themselves (e.g., more muscles) depicting the results obtained if they followed the advice. In this design, users can interact with multiple simulated exercising peers for different fields of expertise (e.g., running, and nutrition). Interactivity with the simulated exercising peer would either be through voice or text messages both possible during and before or after the physical effort. This simulated exercising peer would also adapt the proposed activities to the user’s capabilities and to the weather conditions.

While working on the aesthetics of SEPs, several participants were concerned by the risk of deception. In order to avoid any type of deception, the majority (G1, G2 and G3) used non-human representations for their SEPs. Participants argued that using animals (cf., Figures 4, 5) or cyborgs (cf., Figure 6) instead of a human being would reduce the risks of being deceived. Indeed, these groups (G1 to G3) stated that even with a strong signal (such as the “AI” label on the avatar during the *Contextualization* phase of this experiment, cf., Figure 3) a human avatar would be a possible source of deception because these symbols can be easily missed. Furthermore, these groups reported eerie feelings after competing against Eduardo, arguing that the SEP's avatar was too human-like, as illustrated by [G3, F]: *“Actually, I don’t know, I feel like it is strange [about the SEP avatar]. Personally, I could not look at a complete picture of a person and tell myself it is an AI, it is frustrating, really. It’s frustrating as soon as you know [that it is an AI], […] it is a bit strange to have a photo of a person when you know there is an AI behind it.”* Interestingly, the last group (G4) preferred to have an avatar representing a human in an ideal physical shape (cf., Figure 7) [G4, M]: *“Yes a human clearly”*, [G4, F]: *“It would be more motivating if we see that it is shaped like us […] and that it evolves together with us.”* This group explained that the visual appearance could not lead—by itself, to deception. Participants in G4 used the SEP’s body shape to represent the physical objective they want to attain—thus, modeling an ideal human self to use as motivational objective. However, these participants also stressed the need to have a distinctive visual cue in the proximity of the avatar, to indicate the SEP’s non-human nature (e.g., an “AI” indication on the side).

All designs adopted visual elements that could enable self-identification with the avatar (e.g., non-gendered animal, human shape based on users’ attributes). Our participants explained that identification with their SEP would be key to motivate and engage users in the intervention. In addition, G1 and G2 argued that using animals would ease self-identification as animals are typically looked as gender-neutral beings [G1, F]: *“Personally I would not have used a human […] I would have used an animal, because everyone knows what it is, and can identify with it.”* Interestingly, all groups anticipated that the visual appearance of the SEPs would influence their behavior—a component that we explore in the next section.

Finally, we observed that two groups (G1, G2) designed features that would provide users with rewards. In their designs, the users could receive or buy new personalization accessories for their SEPs (e.g., glasses, t-shirts, collars, etc.) using points rewarded as they exercise and reached their goals. Thus, the rewards effect would be two-fold: encouraging the users through an external stimuli, and enabling the users to further personalize their SEPs visual appearance. This concept is illustrated by one of the participant’s input [G2, M] saying: *“[…] there would be levels to attain, like by doing a certain number of meters per day. Or more like it would be that we do a given number of meters to go from level 3 to level 4 as it provides this sensation of accomplishment […] and with the levels, we would unlock new animals or accessories for the animals, and other things like this.”*

Taken together, these results showed that participants emphasized the need for adaptability of SEPs’ visual appearance and raised concerns about potential deception. Across groups, participants noted that visual appearance could influence their relationship with SEPs, affecting their ability to identify with the agent. Such identification, they suggested, could strengthen connections and encourage engagement. However, they felt that assigning human-like avatar to AI agents like SEPs might evoke eerie feelings and suggested alternative design options to avoid discomfort.

Participants were also aware of contextual and individual preferences in visual design, recommending personalization features to enhance user satisfaction. Some groups also suggested using SEPs visual appearance as a motivational tool, where users could unlock new accessories as rewards for achieving goals, enhancing their engagement with the SEP.

### 5.2 SEPs’ behavior

Participants articulated the behaviors of their SEPs around two roles: *companion* (G1 to G3), and *coach* (G4). As a companion, SEPs would exercise together with their users. Companion SEPs would adjust their physical activity^6^ schedule to the habits of the human companion as if they were to train together. Other designs also proposed that the companion SEPs would be virtually represented next to the users’ avatar while they both walk (cf., Figure 5). In the role of coach, SEPs would not train with their users, but rather act as a human trainer (cf., Figure 7), as further illustrated by G4 during the final group review [G4, F]: *“It would provide us feedback on our performance […] we thought that during the activity it would use audio cues as we can’t use our phone while doing sports in general.”* As suggested by this quote, the SEPs in a coach role would provide suggestions or recommendations to help users reach their goal (e.g., reduce the pace when the SEP sees that the user is tired, or suggesting stretching exercises to do after the effort). In a companion role, a SEP was often represented as a target objective for the user to beat. As participants explained, a companion SEP should leverage social comparison to encourage users. Participants suggested the idea that when exercising with another partner (i.e., the SEP) they would feel encouraged to match or exceed their partner’s activity. Thus, designs from G1, G2, and G3 implicitly sustained the idea that companion SEPs would have to exercise providing an optimal challenge to their human users. In other words, companion SEPs should be capable of tracking and modeling users’ physical activity patterns (e.g., daily step count) to appropriately adjust the difficulty of competitions for their human peers. For example, G3 imagined a SEP that would adopt different behaviors during a week—i.e., setting a very high step count one day, and a lower count another day [G3, M]: *“Like say, every day in a week of five days, you make it so that it [the SEP] is weaker than you, but from a 100th of step, a small margin. And some days you make it augment a little bit [the number of steps done by the SEP] to give you the will to try and outperform it otherwise you are constantly the best […] or it is better with three days out of four, like three days out of four on average we are stronger than it [the SEP], it stays a little bit under [in the number of steps] than us.”* Interestingly, G1 and G2 mentioned that while companion SEPs would respect optimal challenge they should also be capable of adapting their behavior to their visual appearance, i.e., a lion would be faster than a turtle, exemplified by [G2, F]: *“We can choose like, if we want something more in front of us [on the map] to challenge us then we take a certain animal […] certain animal will stay in front of us [on the map] or behind depending on the type of animal.”*.

Group G4 was the only one to provide an example of SEP taking on a coaching role. Rather than acting as a competitor, the SEP would support users in setting and achieving their physical activity goals. Participants emphasized the importance of SEPs’ ability to model user performance (e.g., average steps, daily step count, etc.) to assist in setting goals and providing an optimal challenge (cf., Figure 7).

### 5.3 Communication with SEPs

All the groups designed SEPs that could communicate with their human counterpart. The participants envisioned the use of text (G1 to G3) or voice messages (G4) as a medium to interact with the SEPs. Participants in group G4 thought voice was much more adequate to support communication during the exercise, while the users were busy performing physical activity and therefore interacting with their mobile phone was impractical. All groups desired bidirectional communication with SEPs, [G3, M] mentioned: *“It would be cool that we could also speak with it [the SEP].”* Thus, the users could receive feedback during, or after the effort, but they could also provide feedback to the SEPs. The participants argued that communication from the users to the SEPs could help the SEPs understand the context, e.g., the users are sick and are unable to exercise. For example, G4 emphasized that [G4, M]: *“[…] yeah it [the SEP] could propose activities [or exercises] that correspond to the weather. When it rains we might not go running, but we will do activities at home. […] notify us to do activities when we have not exercised in a while and we could say if we are able to proceed or not.”* Additionally, the messages sent from the users to the SEPs would also help to regulate the goal with respect to the optimal challenge (e.g., the required effort is too high, thus the goal has to be reduced), as mentioned by [G2, F]: *“[…] for when they want a bit more challenge. Like, the animal goes a bit further ahead [on the map] the user can send three types of messages [to their SEP], to continue at the same rhythm, to slow down or to go faster.”* Interestingly, group G1 proposed the use of predefined messages when talking to a SEP in order to avoid *“misunderstandings”* as they identified it as a recurring problem when interacting with CAs.

Furthermore, the participants mentioned that the SEPs role would influence its communication style. Thus, companion SEPs would communicate using friendly informal messages, while the coach SEPs would use a more formal tone. For instance, a participant in G2 expressed a preference against overly formal or directive messages from a SEP acting as a companion. They highlighted the importance of empathy, contrasting it with a “cold”, apathetic entity, like a tree: [G2, M]: *“And also it is important to note that it is more a companion than an expert per se, it gives me some exercising advice but without being like an inanimate tree, it should not give orders like ‘do 15 squats’.”*

Unfortunately, participants were not able to provide examples of messages companion SEPs could send. However, they stated that messages should be encouraging, and supportive to let users feel cared for [G1, F]: *“Every goal achievements it [the SEP] says something like ‘Well done’ […] during the training it keeps sending encouraging messages […] It should never be discouraging.”* Two groups (G1, G2) have also imagined situations where the users would have to care for their SEPs, illustrating it with their interest in getting special accessories obtained as they achieved their goals (as reported in Section 5.1).

These results provide a complete overview of young adults on the use of SEPs as a solution to promote physical activity and support relatedness. The four groups proposed four designs and provided a significant amount of information on different aspects of SEPs’ design, notably:

• Participants stated that SEPs should avoid deception and should provide optimal challenges to their users.
• Participants identified two communication styles for SEPs: companion, and coach, each with their own behaviors, frequencies, and tone.
• Finally, participants wished social interaction could be bidirectional, and that relatedness could also be promoted by enabling users to care for their SEPs.

In Section 6, we discuss these results and the observations made by other scholars.

## 6 Discussion

The designs proposed by our participants highlighted that the risk of deception is a crucial factor for the design of SEPs’ aesthetics, behavior, and communication. For this reason, we structure the discussion in four subsections: deception, appearance, behavior and connectedness. We highlight the key differences in aesthethics, behavior and communication between each SEPs’ roles in Table 5. As we delve into the various components of SEPs, we elaborate on how these elements empower SEPs to encourage users based on the principles of SDT and report a summary in Table 4.

**Table 4:** A summary of SEPs’ features that can promote the basic psychological needs defined by SDT.

Basic psychological need	SEPs features
Autonomy	Personalization
	Independent schedule of SEPs
Competence	Optimal challenge for goal setting
	Optimal challenge for social comparison
Relatedness	Motivational messages
	Virtual presence
	Personalization

**Table 5:** A summary of the key differences in aesthetics, behavior, and communication with respect to SEPs’ roles.

Role	Aesthetics	Behavior	Communication
Coach	Anthropomorphic (e.g., human, humanoid)	Cooperate, Goal-Setting using Optimal Challenge	Formal, recommendations or instructions
Companion	Zoomorphic (e.g., animals)	Compete, (Coopetition), Optimal Challenge	Informal, motivational messages, small-talk

### 6.1 Why we should avoid deception with SEPs design

Participants (3 out of 4 groups) expressed significant concern about possible deception when interacting with peers, fearing they might be unable to distinguish between a SEP and a real human. This concern is amplified as CAs’ capabilities improve (155). Prior research shows that deception within teams reduces relationship quality by decreasing trust and mutuality (130), which could profoundly affect human-agent relationships if agents adopt deceptive appearances, behaviors, or communication skills. Deception has broader implications, as humans often apply similar social factors to human-to-agent relationships as they do to human-to-human interactions (156, 157). Furthermore, agents’ human-like features can disinhibit emotions, fostering bonds like friendship or partnership (158–160). As emotional and social engagement with technology grows, exemplified by phenomena like personification (158, 160), transparency about the entity’s non-human nature becomes crucial. Otherwise, discovering that a teammate or challenger is an AI could leave users feeling tricked, unfairly treated, or tasked with meaningless objectives.

Designing AI agents with human-like features can lead to unintended consequences. For instance, Cowan et al. (161) found that users hesitated in their actions out of concern for hurting the agents’ feelings, potentially affecting intervention outcomes if users’ decisions are influenced by emotional connections with the AI. However, such effects may not persist after the intervention. While human-like features can foster prosocial behaviors (128), they may also provoke deception and feelings of eeriness, as described by Mori (46)’s Uncanny Valley phenomenon. This discomfort is compounded by the inherent tendency of AI development to lean toward deception, as exemplified by the Turing Test’s focus on agents mimicking humans (162). High human-like characteristics, such as anthropomorphic avatars, can lead users to overtrust AI, suspending disbelief and increasing the risk of deception (163–165). Moreover, designing agents that fit users’ idiosyncratic preferences, beliefs, expectations and assumptions about the agents’ capabilities remains challenging (166).

The considerations that are reported so far in this section refer to CAs, but these considerations would not cover the full set of possibilities enabled by SEPs. In addition to conversations, SEPs include features such as simulating to practicing a sport with the user or guiding the user via demonstration, which could also lead to deception if SEPs implement a near-human simulation (e.g., having its own pace and walking habits).

This study generated some recommendations in this regard to avoid this specific type of deception. First, SEPs should not use human avatars (cf., Section 6.2), and this is particularly important if the intervention pairs SEPs and human users in a social comparison context (e.g., in a competition). Second, the implemented communication modality should remain different between human-to-human and human-to-SEP. For instance, humans should only be able to interact with SEPs using predefined prompts. Third, SEPs’ behavior should remain coherent with their visual appearance (cf., Section 6.3). For example, a SEP adopting the visual appearance of a leopard should provide high performance (e.g., larger number of steps during a day or higher speed in a run) than one resembling a sloth.

### 6.2 Aesthetics: the benefits of SEPs’ visual appearance personalization

As noted in the Results section (cf., Section 5), all groups displayed diverse SEPs in visual appearance, highlighting varied user expectations for their digital activity partners. Consequently, it’s crucial to offer users the choice of SEPs’ visual appearance. All groups concurred on the importance of this personalization. Echoing video games literature, personalization can enhance the human-virtual character bond (90) and boost intrinsic motivation by promoting autonomy (85). Although our findings on personalization effects are preliminary, allowing such customization could enhance SEP-based interventions. Our results also support and expand on Birk and Mandryk (86)’s findings regarding game character personalization’s role in increasing engagement and interest in video games, suggesting similar benefits for SEP interventions.

This study provides insights into users’ expectations regarding the visual appearance of a SEP to enhance motivation in physical activity interventions. We found that aesthetic preferences vary depending on the SEP’s role, either as a coach or companion, suggesting that personalization features should match the expected role. For instance, interventions could offer avatar items suitable for coach or companion SEPs. When acting as a coach, some participants preferred SEPs that represent their ideal self, adding complexity to using anthropomorphic visuals which could risk deception. However, studies by Yee et al. (167) and Bessiére et al. (168) suggest that human-like features in avatars, such as height or attractiveness, are more influential than complete human-likeness in enhancing self-perception and well-being. Thus, we recommend incorporating human-like attributes in non-human avatars to avoid deception (cf., Section 6.1). In the companion role, participants favored zoomorphic SEPs for their ability to facilitate self-identification without being constrained by gender, suggesting these avatars effectively support diverse user identities. Past research supports the appeal of zoomorphic avatars among adults and young adults (28, 29) and highlights the benefits of self-representation in virtual environments for psycho-physiological well-being and user satisfaction (91, 169, 170). Offering diverse SEP visual personalization options could therefore enhance user satisfaction and positively impact behavior change interventions through increased self-identification and self-perception, as noted in video game research (167, 171).

Our findings align with existing literature on the effects of virtual entities’ visual appearance, highlighting the importance of self-identification and self-perception with avatars. Human-likeness, however, was not universally supported as an influencing factor due to the risks of deception identified by participants in certain scenarios. These results underscore the necessity for interventions involving SEPs to allow users to personalize their SEPs’ visual appearance. Literature also notes personalization as a valuable opportunity to foster BPNs, indicating its potential effect to also increase engagement in SEP-based interventions. Furthermore, personalization can help tailor SEPs to users’ expectations and preferences, however, require to be aligned with the roles SEPs should adopt to assist users in their behavior change journey. These findings suggest that research on avatar personalization, interpretation, and perception extends beyond video games and virtual worlds, potentially influencing the design of agents like SEPs for behavior change interventions.

We continue our discussions exploring SEPs’ roles and their implications for SEPs’ behavior and communication capabilities.

### 6.3 Behavior: the roles that SEPs can play and the implied behaviors they should adopt

Our results show two specific social roles that SEPs can play, each role having its corresponding expected behavior, i.e., coach or companion. Participants described coach-oriented SEPs’ as supportive digital entities designed to help users achieve defined goals. This is accomplished by influencing users’ thoughts, emotions and actions through conversations—a description aligning with the definition of virtual coaches found in the literature (120, 123, 172–174). Virtual coaches are typically used to provide physical exercise demonstrations (175), to help user set specific goals for their training (120, 123, 172, 173), and advise on health-related matters like nutrition (176). Coach SEPs and users are set to cooperate in order to achieve the users’ defined goals, where the SEP’s purpose is to support motivation and provide technical advice. In the companion role, the SEPs and users are in a competition, thus, becoming a representation of the users’ target to beat. In addition, a group also proposed coopetition as a social strategy engaging companion SEPs and users against other teams composed of humans and SEPs. In both the competition and coopetition strategies, the existence of a performance-contingent reward (i.e., a reward given to the best performing entity) between users, or between users and their SEPs, would likely lead to social comparison. This dynamic was defined in physical activity interventions as “the design that facilitates benchmarking individual’s fitness performance with that of others, and hence provides an opportunity for greater motivation in target behaviours” (99, p. 7), and was also considered as a strategy for physical activity promotion (98, 108). However, research has also demonstrated that social comparison could undermine an individual’s interest in their competitors if the competitors are over-performing them: in this case, the individuals will feel that their competitors are “unreachable” (106). The latter effects are in line with our results: our participants mentioned that social comparison could undermine their motivation in the case where the SEPs would be unbeatable.

Our findings suggest that all SEPs need to provide an optimal challenge. In the coach role, the SEPs would provide an adequate goal for the users to reach by measuring and predicting their physical capabilities. Coach SEPs could further leverage on the goal setting to foster users’ autonomy by letting the users negotiate the next goal. This resonates with most of the virtual coach literature that relies on the agent to advise the user in the goal setting process (120, 123, 172, 173)—emphasizing that coach SEPs should be designed with great attention to optimal challenge so as to set the goal and ensure the promotion of competence. In the companion role, the performance of the SEPs should be adapted to the users’ walking habits and daily number of steps. Thus, companion SEPs would become a fair competitor for the users. Furthermore, our findings suggest that companion SEPs should ensure believability by respecting the users’ expectations emerging from the SEPs’ visual appearance (41, 42, 89). Thus, we observe the SEPs’ believability requirement as an opportunity to let users choose a specific difficulty level or, in other words, the limit of the social comparison gap that could be created between the users and their SEPs. Overall, providing an optimal challenge would let SEPs promote users’ competence BPN as reported by SDT (34).

These results allow extracting two main design implications that fit *RQ2: What are users' expectations for the behavior of simulated exercising peers in promoting motivation for physical activity interventions?*: first, it is crucial to let users choose the specific role of the SEP, namely whether the SEP should behave as coach or companion; second, a SEP should always assess users’ capabilities to set tailored goals that make for an optimal challenge. The role of the SEP will also influence the social strategy that will be adopted between the users and the SEPs. For instance, companion SEPs are to be preferred in a competition or coopetition scenario, while a coach SEP would be the appropriate choice for cooperation.

The choice of the role also has an influence on the design of interactions between users and SEPs and we further analyze on this matter in the next section.

### 6.4 Connectedness: how SEPs’ roles can define their communication styles and their needs

In this study, participants expressed a desire to engage with SEPs during the contextualization phase to understand the entity’s identity and purpose. All groups included a chat feature in their designs, with most favoring textual communication, while one group suggested voice-based interaction for continuous support during exercise. SEPs’ messages were primarily designed to encourage and support users, aligning with research showing such techniques enhance engagement in physical activity interventions (177). However, overly frequent or predictable automated messages can lead to user disengagement (178), emphasizing the need for tailoring messages across dimensions like timing, intent, content, and representation (121). Furthermore, agents should adapt their interactions with the users’ routine (79), and motivation (179) to avoid being ignored.

Participants highlighted bidirectional communication, proposing a prepared prompt-based system to mitigate misunderstandings due to SEPs’ limitations in Natural Language Processing (NLP). Similar mechanisms, as noted by Ashktorab et al. (180), repair misunderstandings common in CAs with restricted NLP capabilities. Such approaches have also been used to minimize errors in agent responses, particularly for rule-based systems that rely on predefined triggers (172, 173). However, while effective, these solutions can limit users’ freedom during interactions, further highlighting distinctions between human-to-human communication and interactions with SEPs.

Participants suggested that SEPs’ communication styles should align with their roles. In the coach role, SEPs would use a formal tone, focusing on recommendations and instructions. As companions, SEPs would adopt a more informal, friendly style, with frequent messages, including small talk and motivational interactions. This contrast may reflect participants’ experiences with human counterparts in similar roles. This resonates with the literature on companionship that defines it as a social relationship characterized by intimacy, shared activities, and emotional connection (181–184), whereas coaches are often viewed as professional service providers (185), leading to expectations of a more structured interaction.

These findings align with previous research showing that CAs’ communication style similarity enhances users’ sense of presence (186). Informal communication by companion SEPs may increase perceived relatedness and companionship, while coach SEPs focus on optimizing user performance. However, neither our findings nor existing studies clarify how relatedness differs between these roles. While this study focused on the coach and companion roles, SEPs could also serve broader roles in contexts like family dynamics, fostering relatedness and integration in social settings (187).

Participants primarily designed SEPs to support users by nurturing their relationship with them. However, our findings suggest that companion SEPs could also receive support from users, reinforcing the bond rather than solely motivating through nurturance. These observations are consistent with prior research showing that humans form relationships and care for virtual entities (28, 29, 188). Additionally, participants proposed using rewards not for traditional self-rewards (e.g., badges or trophies) but to unlock visual accessories for SEPs, fostering care and strengthening the connection.

These findings resonate with studies on virtual pets, such as the iconic Tamagotchi,^7^ described as “creatures from another planet that needed human nurturance, both physical and emotional” (189, p. 506). Although Tamagotchis created strong bonds, they were often discarded when users lost interest (190). This risk also applies to SEPs, though it may be mitigated as SEPs focus on supporting users’ behavior change, rather than serving solely as entertainment tools.

Based on empirical results and participants’ designs, we identified key features to answer *RQ3: What are the necessary features that would make users feel connected with simulated peers?* First, bidirectional communication should be enabled, with predefined prompts to minimize SEPs’ misunderstandings. However, this may evolve as new NLP techniques, such as the mainstream adoption of ChatGPT, show unprecedented results in language and intent understanding (191). Second, our findings suggest that users seek believable agents, which requires clear role definitions for SEPs. Beyond visual appearance and behavior, communication capabilities should align with the social role SEPs assume in the intervention. This extends the theory of agent believability, showing that user expectations are influenced by the roles SEPs are designed to play. Finally, we advocate for integrating gamification, where rewards can personalize SEPs’ appearance. This approach would make goal achievement more meaningful, as users perceive rewards as virtual gifts for their SEPs, potentially strengthening bonds and promoting relatedness, similar to dynamics observed on digital platforms and in games (192, 193).

### 6.5 Summary of design implications

As discussed in the previous subsections, this participatory design study allowed for the identification of five key implications for the design of SEPs that we summarize in this list:

1. **Implement distinctive visual cues:** Ensure SEPs have clear visual markers (e.g., a unique color scheme, abstract or robotic features) that differentiate them from humans in order to avoid risks of deception. For instance, use non-human designs or other distinctly artificial characteristics;
2. **Define and align SEPs’ roles:** Clearly define the social role of each SEP (e.g., coach or companion) before starting the design process. Shape their behavior and communication style to match this role. For example, a coach SEP could use motivational language and structured feedback, while a companion SEP might use more casual, friendly interactions;
3. **Enable personalization:** Allow users to customize their SEP’s visual appearance to reflect their preferences and ideal selves. Provide a variety of customization options such as body type, attire, and accessories that align with the SEP’s role (e.g., sports’ expert gear for a coach SEP);
4. **Personalize challenges:** Design SEPs to assess users’ physical activity levels using wearable devices or app integrations. Based on this assessment, personalize the activity goals to provide an optimal challenge. For example, gradually increase step targets as users’ fitness improves; and
5. **Facilitate bidirectional communication:** Create communication features that allow users and SEPs to interact in a meaningful way. Integrate options for users to send and receive virtual gifts or messages, fostering a sense of mutual care. For instance, SEPs could send encouraging messages or virtual rewards after users achieve their goals, and users could thank their SEPs for support or adjust SEPs’ behavior.

### 6.6 Limitations of our approach

Our study was conducted with several limitations. Firstly, the design of SEPs, like other agent designs, may depend on various social factors and demographics, such as region, beliefs, culture, age, or gender of the participants. While we made efforts to select participants with diverse backgrounds and aimed for equal gender representation, our findings cannot be generalized beyond the young adult population in this region. Achieving broader generalization within this demographic would require additional participatory design sessions across various regions worldwide. Furthermore, our participatory design cohort consisted primarily of young adults, as this was the specific target group of our research. While involving teenagers or older adults might have led to distinct prototypes and perspectives on SEPs, our central objective was to explore the needs and expectations of young adults, and our study was not designed to address other age groups.

Secondly, we relied on a pre-existing mobile application to contextualize our participants, while we gained time using it, it imposed certain limitations. We could only represent the SEP through an avatar and a step counter indication displayed on a leaderboard. Moreover, participants could see their teammates (session participants) on the leaderboard, possibly influencing social comparisons. Some smartphones already stored step counts during the day, and our mobile application synchronized these counts, providing certain participants with an advantage. While the social comparison didn’t hinder motivation for activity performance, competing against such a high-performance teammate prompted remarks by some participants. To address such emotional states, we limited our questions to the SEP’s performance compared to theirs, allowing them to focus solely on the design of the SEP.

Thirdly, the leaderboard structure inherently placed participants in a competitive position, not letting them experience cooperation or coopetition. This situation might have impacted the SEP’s design concerning the chosen social strategy. In an attempt to alleviate this limitation, we emphasized the behavior of the SEP rather than other group members to the participants.

## 7 Conclusion

We have involved a total of 16 young adults in participatory design sessions to gain insights on the design of new AI agents to support physical activity and foster relatedness. By analyzing scientific literature and adopting a user-centered approach, we have distilled a comprehensive list of design implications for SEPs, encompassing three main components: aesthetics, behavior, and communication. These design implications contribute to the field of HCI, offering insights into how to address the potential challenges of deception that can arise when users struggle to distinguish between human peers and AI agents. Participants highlighted that attention to SEPs’ visual appearance could facilitate self-identification, and emphasized its importance in defining SEPs’ behavior and communication styles to ensure believable and effective agents.

We have also identified SEPs’ potential to foster motivation for physical activity by effectively addressing SDT’s BPNs. Of particular significance, we elaborate on the implementation of optimal challenge with SEPs as an additional means to create fair objectives to support users in their physical activity behavior change journey.

Finally, the ongoing advancements in AI agency, exemplified by the emergence of Large Language Models (LLMs), hold the promise of a bright future for the development of SEPs with enhanced capabilities to support individuals in their daily physical activity endeavors. We hope that the presented study will contribute to the design of technological interventions that could empower users and allow them to have a healthier lifestyle.

Looking ahead, future work will delve deeper into the practical implementation of these design implications. This will involve incorporating SEPs into longitudinal experiments to explore how users interact with these agents and interpret the integration of their aesthetics, behavior, and communication skills. Additionally, future research should address the potential ethical issues associated with agents that possess human-like appearances and implement LLMs’ advanced conversational skills, which could expose users to Uncanny Valley or deception effects. By continuing to refine and test SEPs, we aim to enhance their effectiveness and ensure they provide meaningful support in promoting healthier lifestyles.

## Data Availability

The datasets presented in this study can be found in online repositories. The names of the repository/repositories and accession number(s) can be found below: https://doi.org/10.17605/OSF.IO/9T3CM.

## Footnotes

^1^Young adulthood is set as a period spanning from 18 to 25 years old (2, 6). Most researchers define young adulthood as a transitional period where individuals *“participate in self-exploration to cultivate a personal identity and belief system, all the while gaining independence and autonomy”* (6, p.3) such as is the case of university students (3).

^2^See https://doi.org/10.17605/OSF.IO/9T3CM, last accessed November 2024.

^3^See https://www.mypacer.com/, last accessed November 2024.

^4^See https://doi.org/10.17605/OSF.IO/9T3CM, last accessed November 2024.

^5^See https://doi.org/10.17605/OSF.IO/9T3CM, last accessed November 2024.

^6^In our context this means the SEP would record steps like if it was a regular user.

^7^See https://tamagotchi-official.com/us/, last accessed November 2024.
